# Antimicrobial Resistance Profiles of Coagulase-Negative Staphylococci in Community-Based Healthy Individuals in Germany

**DOI:** 10.3389/fpubh.2021.684456

**Published:** 2021-06-17

**Authors:** Gabriella Marincola, Olivia Liong, Christoph Schoen, Alaa Abouelfetouh, Aisha Hamdy, Freya D. R. Wencker, Tessa Marciniak, Karsten Becker, Robin Köck, Wilma Ziebuhr

**Affiliations:** ^1^Institute of Molecular Infection Biology, University of Würzburg, Würzburg, Germany; ^2^Institute of Hygiene and Microbiology, University of Würzburg, Würzburg, Germany; ^3^Department of Microbiology and Immunology, Faculty of Pharmacy, Alexandria University, Alexandria, Egypt; ^4^Department of Microbiology and Immunology, Faculty of Pharmacy, AlAlamein International University, AlAlamein, Egypt; ^5^Helmholtz Institute for RNA-based Infection Research (HIRI), Helmholtz Centre for Infection Research (HZI), Würzburg, Germany; ^6^Friedrich Loeffler-Institute of Medical Microbiology, University Medicine Greifswald, Greifswald, Germany; ^7^Deutsches Rotes Kreuz (DRK) Kliniken Berlin, Berlin, Germany; ^8^Institute of Hygiene, University Hospital Münster, Münster, Germany

**Keywords:** coagulase-negative staphylococci, antimicrobial resistance, One Health, community settings, Germany

## Abstract

Coagulase-negative staphylococci (CoNS) are common opportunistic pathogens, but also ubiquitous human and animal commensals. Infection-associated CoNS from healthcare environments are typically characterized by pronounced antimicrobial resistance (AMR) including both methicillin- and multidrug-resistant isolates. Less is known about AMR patterns of CoNS colonizing the general population. Here we report on AMR in commensal CoNS recovered from 117 non-hospitalized volunteers in a region of Germany with a high livestock density. Among the 69 individuals colonized with CoNS, 29 had reported contacts to either companion or farm animals. CoNS were selectively cultivated from nasal swabs, followed by species definition by 16S rDNA sequencing and routine antibiotic susceptibility testing. Isolates displaying phenotypic AMR were further tested by PCR for presence of selected AMR genes. A total of 127 CoNS were isolated and *Staphylococcus epidermidis* (75%) was the most common CoNS species identified. Nine isolates (7%) were methicillin-resistant (MR) and carried the *mecA* gene, with seven individuals (10%) being colonized with at least one MR-CoNS isolate. While resistance against gentamicin, phenicols and spectinomycin was rare, high resistance rates were found against tetracycline (39%), erythromycin (33%) and fusidic acid (24%). In the majority of isolates, phenotypic resistance could be associated with corresponding AMR gene detection. Multidrug-resistance (MDR) was observed in 23% (29/127) of the isolates, with 33% (23/69) of the individuals being colonized with MDR-CoNS. The combined data suggest that MR- and MDR-CoNS are present in the community, with previous animal contact not significantly influencing the risk of becoming colonized with such isolates.

## Introduction

Antimicrobial resistance (AMR) in bacteria is an increasing public health issue jeopardizing many achievements of modern medicine (1). Accordingly, monitoring the resistance situation in major human and veterinary pathogens such as enterobacteria, non-fermenters or *Staphylococcus aureus* is in the focus of surveillance programs. Far less attention however is paid to commensal, low pathogenic and environmental microorganisms, which may carry AMR genes as well. In this context, commensal and environmental bacteria are considered to play a role as putative AMR gene reservoirs that may fuel the resistance gene pool of more pathogenic bacteria through horizontal gene transfer (HGT) (2–4). Also, under certain conditions, these bacteria may be selected and emerge as opportunistic pathogens in their own right. A prime example for the dual role of commensals are coagulase-negative staphylococci (CoNS), which form a significant part of the skin and mucosa microbiota of warm blooded hosts, but also represent classical opportunistic pathogens that have been established as common causes of numerous healthcare-associated infections (5–7). Nosocomial CoNS are particularly notorious for readily acquiring numerous resistance traits, resulting in (multidrug-)resistance toward many commonly used antimicrobials. In addition, some species (e.g., *Staphylococcus epidermidis*) are capable of forming biofilms on indwelling medical devices, making CoNS infections sometimes extremely difficult to treat (8). While the detection and spread of multidrug-resistant (MDR) CoNS in hospital settings is well-documented (5, 9), we currently have less information on the resistance situation in CoNS outside of medical facilities. Previously, we performed a study on AMR in CoNS recovered from dust and manure samples in pig farms with a previous history of livestock-associated (LA) methicillin-resistant *S. aureus* (MRSA) detection (10). Unexpectedly, we found high AMR rates in CoNS from environmental samples, including resistance traits against last resort antibiotics such as oxazolidinones and lipopeptides. The reason(s) for the high multidrug-resistance rate in this distinct CoNS collection remained elusive. So, it was speculated that the selective pressure by antibiotics, commonly used in industrialized pig farming, might have favored AMR development. Also, contact of these dust- and manure-derived CoNS with soil microorganisms and their intrinsic resistance gene pool was hypothesized to have facilitated AMR acquisition (10). Finally, it is conceivable that, regardless of the ecological niche they are residing in, CoNS might be generally prone to increased AMR carriage. In order to shed more light on the presence and spread of AMR in CoNS, we currently aim at investigating CoNS from various ecological origins. In this report, we focus on the AMR profiles of human commensal CoNS isolates in non-hospitalized volunteers from the general population in Germany. The isolates were recovered from nasal swabs obtained in a previous cohort study on nasal colonization by important human bacterial pathogens (11, 12). Using standard microbiological methods, we assessed the species distribution and AMR profiles of 127 human commensal CoNS isolates. As the cohort study was performed in a geographic region of Germany with high livestock and industrialized farming intensity, we also asked the question whether or not contact with animals may represent a risk factor for individual AMR-CoNS carriage. Together, the analysis revealed that AMR is widespread among human commensal CoNS, many of which detected as MDR resistant isolates, with animal contact not significantly influencing individual AMR carriage.

## Methods

### Sample Isolation, Isolate Recovery, and Species Identification

For the analysis, we referred to a previous cohort study in which 1,878 nasal swabs were obtained from non-hospitalized volunteers from the German general population (11, 12). Recruitment was done by asking persons for their agreement to voluntarily participate in the study; written informed consent was obtained prior to enrolment and ethical clearance was granted by the institutional review board of the Westphalian Wilhelms-University Münster (no. 2006-268-f-S) (11). For CoNS recovery, 65 nasal swabs from persons without animal contact and 52 with animal contact (five veterinarians, 11 farmers and 36 pet owners) were randomly selected for the analysis. Samples were recovered through enrichment in LB broth for 6 h at 37°C. Dilutions of the cultures were plated onto Columbia colistin-aztreonam blood agar (CAP, Oxoid, Germany) and incubated for 24 h at 37°C to select for Gram-positive bacteria and to obtain single colonies. The next day, six randomly selected colonies from each nasal swab were picked and patched onto chromogenic medium to differentiate between methicillin-resistant (MR) and methicillin-susceptible (MS) CoNS (CAMSA/MPK, Medco Diagnostika, Germany). Species identification of the isolates was done by 16S rDNA locus sequencing after PCR amplification using the primers listed in Supplementary Table 1. By this approach, a total of 176 CoNS arising from 69 nasal swabs were obtained for further analysis.

### Antimicrobial Susceptibility Testing

MICs for oxacillin (OXA), gentamicin (GEN), levofloxacin (LEV), erythromycin (ERY), clindamycin (CLI), linezolid (LNZ), daptomycin (DAP), teicoplanin (TEC), vancomycin (VAN), tetracycline (TET), tigecycline (TIG), fosfomycin (FOS), fusidic acid (FUS), rifampicin (RIF), trimethoprim/sulfamethoxazole (TMP-SMX) were determined using the VITEK2 system (bioMérieux Deutschland GmbH, Nürtingen) according to standard procedures provided by the manufacturer (Vitek Card AST-P654). MIC results were evaluated through the Advanced Expert System (AESTM) according to EUCAST guidelines^1^ and clinical breakpoints for CoNS. Isolates arising from the same nasal swab, which were the same species and had the same VITEK antibiogram were considered as duplicates and only one isolate was included into further analyses. Antibiotic susceptibilities for apramycin (APR), spectinomycin (SPC), florfenicol (FFC), chloramphenicol (CM) and quinupristin-dalfopristin (QD) were performed by agar disk diffusion assays using disks with 15, 100, 30, 30, and 15 μg of the respective antimicrobial agent according to EUCAST guidelines. As no interpretive criteria applicable to staphylococci are available for APR, SPC, and FFC, inhibition zone distributions were determined. Isolates displaying reduced zone diameters were further tested by molecular analysis for the presence of the respective resistance genes (see below). Isolates which harbored a respective resistance gene were considered as resistant, even in absence of available interpretive criteria for the antibiotic.

### Molecular Analysis of Resistance

CoNS displaying a resistant phenotype based on the VITEK or disk diffusion analyses were tested by PCR for presence of the respective resistance genes [oxacillin: *mecA, mecB, mecC*; chloramphenicol: *cat194, cat221, cat223*; florfenicol: *fexA* and *fexB*; spectinomycin: *spc, spd* and *spw*; apramycin: *apmA*; fusidic acid: *fusB, fusD, fusC*; gentamicin; *aac(6')/aph(2”)* and *aadD*; erythromycin: *ermA, ermB* and *ermC*; tetracycline: *tetK/L* and *tetM*], using the primers and conditions listed in Supplementary Table 1. Thus, isolates were cultured on CAP sheep blood agar and DNA was extracted using the NucleoSpin Tissue Kit (Macherey-Nagel, #740952) according to the manufacturer's protocol with the addition of 15 μl of lysostaphin (2 mg/ml) to the lysis buffer. Primers used for 16S rDNA amplification were included in each PCR reaction as a control for gDNA template integrity.

### Statistical Analysis

Whenever appropriate, contingency analyses were performed using Fisher's exact test by employing the GraphPad Prism software package. Differences with *p* < 0.01 were considered statistically significant.

## Results

### CoNS Recovery and Species Determination

**Figure 1:**
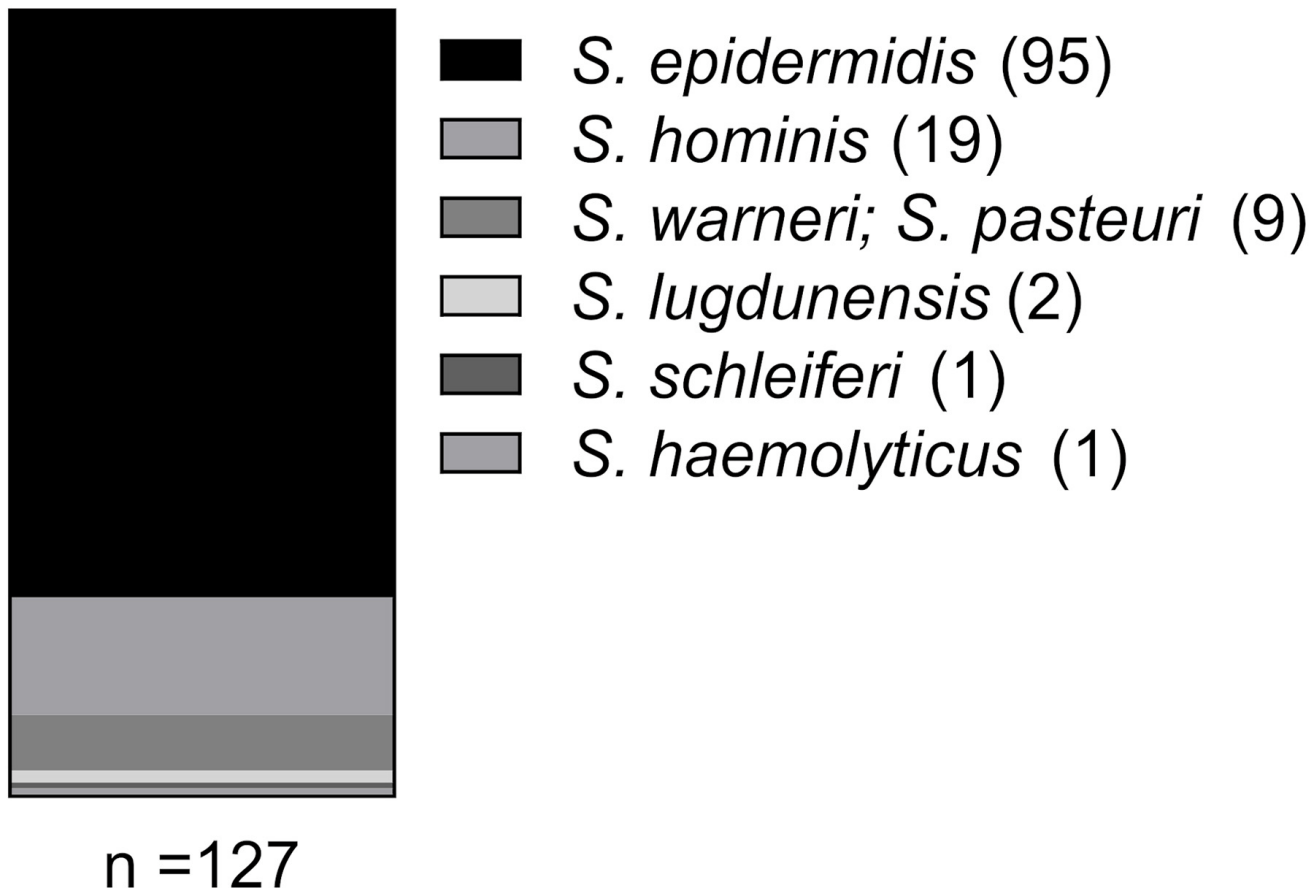
Species distribution among the CoNS isolates as identified by 16S rDNA locus sequence analysis.

For CoNS recovery, 65 nasal swabs from persons without animal contact and 52 with animal contact (five veterinarians, 11 farmers and 36 pet owners) were randomly selected for the analysis. 176 CoNS (arising from 69 nasal swabs) were initially picked from selective media as described in Methods. Next, species and antibiograms of the 176 CoNS isolates were assessed. Isolates arising from the same nasal swab and displaying identical species and resistance profiles were considered as duplicates, with only one isolate being subjected to further analysis. This led to a final pool of 127 CoNS isolates obtained from 69 nasal swabs. In 34 swabs growth of only one CoNS isolate was detectable, while 35 samples exhibited simultaneous growth of more than one CoNS isolate (18 swabs displayed two CoNS isolates, 12 swabs showed three CoNS isolates, four swabs had four CoNS isolates and in one swab we found five different CoNS isolates). Species determination by 16S rDNA locus sequencing identified 75% of the isolates as *S. epidermidis* (95/127). The remaining 32 isolates represented five additional species (Figure 1). *S. epidermidis* was detected in the majority of swabs (61/69; 88%). In the 35 nasal swabs in which more than one CoNS isolate was recovered, at least one *S. epidermidis* isolate was present in the majority of samples (32/35; 91%), while the species was absent in only three swabs (3/35; 9%).

### Antibiotic Susceptibility and Resistance Genes Detection

**Figure 2:**
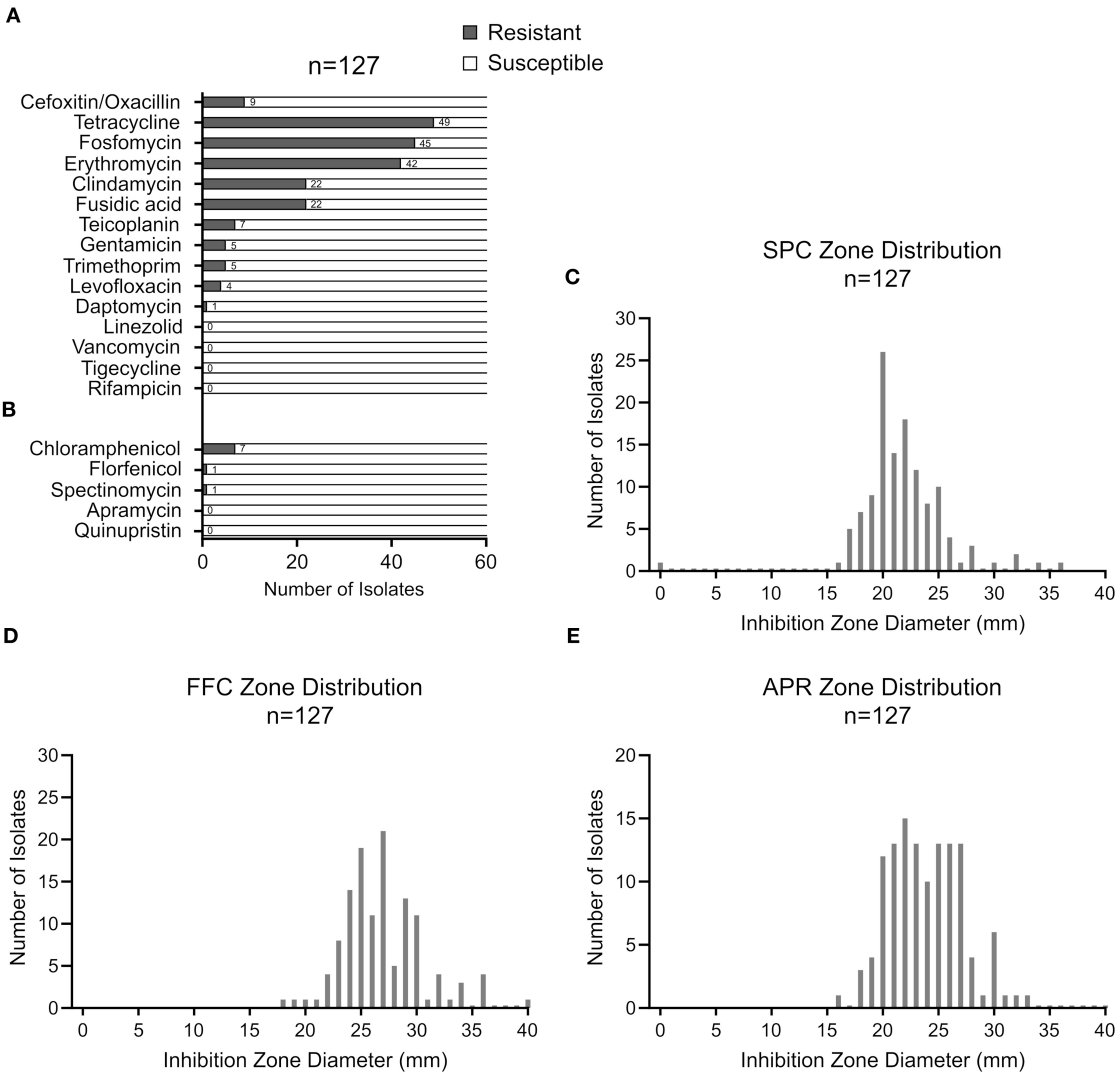
CoNS resistance profiles assessed by VITEK2 **(A)** or by agar disk diffusion **(B)**. Inhibition zone diameter distributions for spectinomycin **(C)**, florfenicol **(D)** and apramycin **(E)** with 100, 30, and 15 μg of the respective antimicrobial agent, respectively, according to EUCAST guidelines.

**Figure 3:**
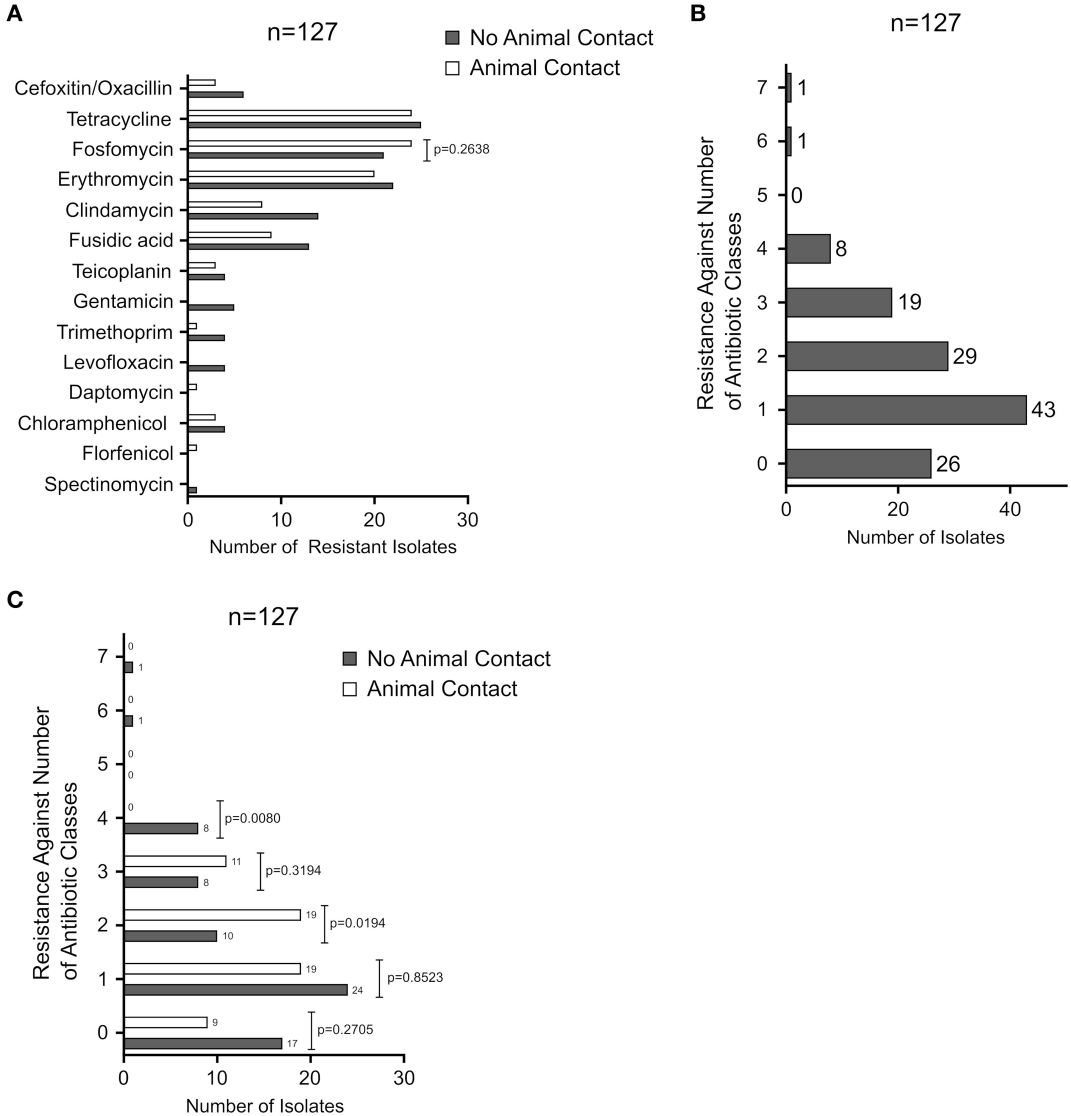
**(A)** Comparison of the number of resistant isolates arising from nasal swabs of individuals with animal contact (white bars) or without animal contact (gray bars). **(B)** Analysis of multidrug-resistant isolates. The number of resistant isolates is plotted against the simultaneous resistance toward 0–7 antibiotic classes. **(C)** Comparison of the number of multidrug-resistant isolates arising from nasal swabs of individuals with animal contact (white bars) or without animal contact (gray bars). Contingency analysis in **(A,B)** was performed using Fisher's Exact Test by employing the GraphPad Prism software package.

The resistance phenotypes and AMR genes detected among the 127 CoNS isolates are summarized in Figure 2 and Table 1. Methicillin (oxacillin) resistance occurred in 7% (9/127) of the isolates (seven *S. epidermidis* and two *S. hominis*), with all isolates carrying the methicillin resistance conferring *mecA* gene (Table 1). None of the MR-CoNS harbored *mecB* or *mecC*. The most abundant resistance phenotypes in the collection were found toward tetracycline (39%; 49/127), fosfomycin (35%; 45/127), erythromycin (33%; 42/127) and fusidic acid (17%; 22/127) (Figure 2A). Gentamicin resistance occurred in five isolates (4%) all harboring the aminoglycoside resistance-mediating *aac-aph* gene. Except for one isolate, tetracycline resistance was conferred either by *tetK/L* (44/49) or *tetM* (4/49) (Table 1). With respect to erythromycin, all 42 resistant isolates harbored at least one of the macrolide resistance genes tested. The most abundant gene was *ermA* (24/42), followed by *ermC* (17/42) and *ermB* (11/42) (Table 1). Interestingly, eight isolates carried more than one of the macrolide resistance genes. Among the 22 fusidic acid resistant isolates, 19 tested positive for *fusB* and three for *fusC* (Table 1). For the antibiotics listed in Figure 2B, agar disk diffusion tests were performed. Based on zone diameter breakpoint definitions for quinupristin-dalfopristin (i.e., *R* <18 mm), all isolates were found to be susceptible to the streptogramin combination (Figure 2B). For spectinomycin, florfenicol, and apramycin no clinical interpretive criteria are currently defined for CoNS. Therefore, inhibition zone diameter distributions were determined and isolates displaying reduced inhibition zones were tested for resistance gene presence (Figures 2C–E). For spectinomycin, one isolate displayed no inhibition zone and carried the spectinomycin resistance-mediating *spc* gene (Table 1). Of the two isolates with reduced zone diameters toward florfenicol (<20 mm) one was found to harbor *fexB* (Table 1). Four isolates with reduced zone diameters (<19 mm) toward apramycin, were analyzed for the presence of *apmA*, but all lacked the resistance gene (Figure 2E). Finally, seven isolates displaying reduced chloramphenicol inhibition zone diameters (≤18 mm) were tested for the *cat* genes, revealing the detection of *cat194* in all isolates (Table 1). Between individuals with and without animal contact, no statistically significant differences were recorded regarding AMR-CoNS carriage, although a slightly increased tendency for the detection of fosfomycin resistant isolates was observed in persons with reported animal contacts (Figure 3A).

**Table 1:** Antimicrobial resistance gene detection among phenotypically resistant CoNS isolates.

**AMR resistance phenotype**	**Number (% of resistant isolates)**	**Species as for 16S (number of resistant isolates)**	**AMR PCR gene detection in resistant isolates**
Oxacillin (^*^)	9/127 (7%)	*S. epidermidis* (7) *S. hominis* (2)	*mecA* (9/9) *mecB* (0/9) *mecC* (0/9)
Tetracycline (^*^)	49/127 (39%)	*S. epidermidis* (37) *S. hominis* (7) *S. haemolyticus* (1) *S. warneri/S. pasteuri* (4)	*tetKL* (44/49) *tetM* (4/49) (4 *S. epidermidis*)
Erythromycin (^*^)	42/127 (33%)	*S. epidermidis* (34) *S. hominis* (6) *S. haemolyticus* (1) *S. warneri/S. pasteuri* (1)	*ermA* (24/42) (19 *S. epidermidis*, 5 *S. hominis*) *ermC* (17/42) (14 *S. epidermidis*, 2 *S. hominis*, 1 *S. warneri/S. pasteuri*) *ermB* (11/42) (8 *S. epidermidis*, 2 *S. hominis*, 1 *S. haemolyticus*)
Fusidic acid (^*^)	30/127 (24%)	*S. epidermidis* (18) *S. hominis* (8) *S. warneri/S. pasteuri* (4)	*fusB* (20/30) *fusC* (3/30) (3 *S. hominis*)
Gentamicin (^*^)	5/127 (4%)	*S. epidermidis* (3) *S. hominis* (1) *S. haemolyticus* (1)	*aac(6')/aph(2”)* (5/5) *aadD* (0/5)
Chloramphenicol (^**^)	7/127 (6%)	*S. epidermidis* (5) *S. hominis* (1) *S. schleiferi* (1)	*cat194* (7/7) *cat221* (0/7) *cat223* (0/7)
Florfenicol (^**^)	1/127 (<1%)	*S. epidermidis* (1)	*fexB* (1/1) *fexA* (0/1)
Spectinomycin (^**^)	1/127 (<1%)	*S. hominis* (1)	*spc* (1/1) *spd* (0/1) *spw* (0/1)

The resistance phenotype was assessed by VITEK (^*^) or by agar disk diffusion assay (^**^). For spectinomycin and florfenicol, strains were considered resistant in the presence of the respective AMR genes (see text for details). Species identification was performed by 16S rDNA loci sequencing; AMR genes were detected by PCR using the primers listed in Supplementary Table 1.

### CoNS Multidrug-Resistance

Multidrug-resistance (defined as insusceptibility toward at least three antimicrobial classes) is a known hallmark and major issue particularly in nosocomial and infection-associated CoNS (5, 9). Analysis of our community-obtained commensal CoNS collection revealed that 20% (26/127) of the isolates were fully susceptible to all antimicrobials tested, while 57% (72/127) exhibited resistance toward one (43/127) or two (29/127) of the antibiotics tested (Figure 3B). Multidrug-resistance was recorded in 23% (29/127) of all isolates, from which 15% (19/127) displayed resistance against three and 6% (8/127) against four antibiotics. Two isolates were simultaneously resistant to six and seven of the antibiotics, respectively (Figure 3B). As shown in Figure 3C, previous animal contact of the volunteers did not significantly influence individual multidrug-resistant CoNS carriage (Figure 3C).

### Individual Colonization by Resistant CoNS

Among the 69 CoNS-carrying individuals (40 with no and 29 with animal contact), seven (10%) were colonized by at least one *mecA*-positive MR-CoNS. The carriage rates were 5/40 and 2/29 for individuals with no or with animal contact, respectively (Fisher's Exact Test, *p* = 0.69, ns). Carriage of fully susceptible and of strains resistant to 1–2 antibiotic classes occurred in 12 (17%) and 34 (49%) of the 69 persons, respectively, from which 4 (6%) and 16 (23%) had previous animal contact (Fisher's Exact Test, *p* = 0.51, ns). Colonization by MDR-CoNS was overall detected in 23 individuals (33%). Among these, there were 9/29 individuals with animal contact vs. 14/40 without animal contact (Fisher Exact Test, *p* = 0.80, ns). The combined data suggest that MR- and MDR-CoNS are widely disseminated in the community with animal contact not significantly influencing the risk of becoming colonized by such isolates.

## Discussion

AMR in bacterial pathogens continues to represent a major challenge for infection control. In order to tackle the problem holistically, the One Health concept, which takes humans, domestic and wild animals and the environment equally into account, is currently being pursued. Across these sectors, the approach also includes commensal and environmental bacteria to assess the risk factors for AMR development in pathogens (13). CoNS are typical skin and mucosa commensals which share the same ecological niche in the human anterior nares with *S. aureus* and many other bacteria (5, 7, 11), providing CoNS ample opportunity for horizontal gene transfer and the exchange of resistance genes (14). Indeed, CoNS have been identified as reservoirs and source of resistance traits that are transferred across the *Staphylococcaceae* family (15), including resistance genes against last resort antibiotics such as linezolid or daptomycin (10, 16). Across the geographic regions and infection sites, high resistance rates are common and typical among CoNS from health care settings (9, 17–20). In this report, we show that methicillin- and multidrug- resistant CoNS are also present in the community in healthy non-hospitalized volunteers. In staphylococci, methicillin/oxacillin resistance is of particular interest. It is mainly mediated by *mecA* (encoding an alternative penicillin-binding protein) located on transferrable SCC*mec* genomic elements whose genetic origins have been associated with *Staphylococcus sciuri* (now *Mammaliicoccus sciuri*) (21–24) and *macrococcal* species (25). SCC*mec* elements readily integrate other mobile genetic elements, and in addition to beta-lactam insusceptibility, they may therefore confer resistance to unrelated antibiotic classes too (15), making *mecA*-carriage a marker for multidrug- resistant isolates as well. Moreover, co-selection processes may favor the manifestation of multidrug resistance. In our study, *mecA* detection among the CoNS isolates was low (i.e., 7%) and only seven of the 69 individuals tested (10%) were colonized by at least one MR-CoNS isolate. These low *mecA* detection rates are in good agreement with previous reports on community-acquired commensal CoNS in Europe (26–28); but numbers may vary considerably in studies conducted in other geographic regions, with MR-CoNS rates ranging between 16 and 50% (29–33). We also noticed relatively high resistance rates toward tetracycline (39% 49/127) and erythromycin (33%; 42/127) in the sample (Figure 2). Macrolides are among the antibiotics most frequently prescribed on an outpatient basis and tetracyclines are commonly used in veterinary medicine. It is therefore well conceivable that the frequent detection of these resistances might be associated with a high selective pressure imposed by these antibiotics. However, as we lack concrete data on antibiotic consumption, this is currently mere speculation. We further found that the majority of isolates (i.e., 77%) are either completely susceptible to the tested antibiotics (20%, 26/127) or show resistance to a maximum of two antibiotic classes (57%, 72/127) (Figure 3B). Of note, however, in about a quarter of the strains (i.e., 23%), we detected multidrug-resistance toward three or more antibiotics, with two isolates even displaying simultaneous resistance against six and seven antibiotics, respectively (Figure 3B). Fortunately, we did not find any resistance to newer antibiotics such as linezolid or daptomycin in our sample, as described earlier in CoNS from animal and human sources (10, 16). Our results are supported by other studies reporting co- and multidrug-resistance not only in CoNS from humans but also among isolates from animals and the environment (34–41). We interpret these findings as an alarming signal for the continued introduction of (multidrug) resistant CoNS isolates into habitats outside of hospitals. Since the selective pressure by antibiotics is the main driving force behind the emergence and maintenance of resistant bacteria, it is tempting to speculate that the ubiquitous use of antimicrobial agents in human and veterinary medicine as well as in agriculture is the key factor behind this worrying development. In addition to their accepted role as resistance gene reservoirs for the more pathogenic *S. aureus* (15, 42, 43), MDR-CoNS selected in the community may pose a risk as opportunistic pathogens in immunocompromised patients when such strains are transferred into hospitals, highlighting the need for effective AMR surveillance also outside of the medical sector. In this respect, both farm and wild animals as well as pets are increasingly recognized as so far neglected source of MDR bacteria, including CoNS (40, 41, 44–50). Another question that we therefore wanted to answer with the study addressed the possible influence of animal contact on colonization rates with MDR-CoNS isolates. Interestingly, the data suggest that animal contact (both to farm and companion animals) did not increase the risk of becoming colonized by such isolates. However, our study was carried out in a region in northwest Germany with high livestock density and a previously proven LA-MRSA prevalence (11). It is well conceivable that the general population in this area is already increasingly exposed to resistant CoNS from animal husbandry, which would make it more difficult to reveal putative animal contact effects. A comparison of the CoNS resistance situation in a comparable region without extensive livestock husbandry would be helpful in order to finally answer this question. Together, the study shows that CoNS, including MDR strains, are present in the community, irrespective of a history of animal contact.

## Data Availability Statement

The original contributions presented in the study are included in the article/Supplementary Material, further inquiries can be directed to the corresponding author/s.

## Ethics Statement

The studies involving human participants were reviewed and approved by Westphalian Wilhelms-University Münster (nr. 2006-268-f-S). The patients/participants provided their written informed consent to participate in this study.

## Author Contributions

GM, CS, AA, KB, RK, and WZ conceived and designed the experiments. GM and OL performed the experiments. GM, OL, AH, AA, FW, TM, and WZ analyzed the data. GM and WZ wrote the manuscript. All authors contributed to the article and approved the submitted version.

## Conflict of Interest

The authors declare that the research was conducted in the absence of any commercial or financial relationships that could be construed as a potential conflict of interest.

## Footnotes

^1^http://www.eucast.org.
