# Supplementary material for: Antimicrobial Resistance Profiles of Coagulase-Negative Staphylococci in Community-Based Healthy Individuals in Germany
Source: Front Public Health. 2021 Jun 17;9:684456. doi: 10.3389/fpubh.2021.684456 (PMC8247762; doi:10.3389/fpubh.2021.684456)
Supplement: Supplementary file 1 [file Data_Sheet_1.PDF]

Supplementary Table 1. List of oligonucleotide sequences and conditions used for amplification of selected antimicrobial resistance genes and 16S rDNA gene loci

Gene	Resistance phenotype	Annealing temperature<br>Amplicon size	Oligonucleotide primer sequence (5’-3’)	Reference
16S rRNA	n.a.	55 °C	16S for: TCCAGATTACAACTTCACCAGG	
		1500 bp	16S rev: GTTCTGCAGTACCGGATTTGC	
mecA	Oxacillin	55 °C	GM155: TCCAGATTACAACTTCACCAGG	(Milheiriço et al., 2007)
		624 bp	GM116: GTTCTGCAGTACCGGATTTGC	(Stegger et al., 2012a)
mecB	Oxacillin	53 °C	GM047: TTAACATATACACCCGCTTG	(Becker et al., 2018)
		2264 bp	GM048: TAAAGTTCATTAGGCACCTCC	(Becker et al., 2018)
mecC	Oxacillin	52 °C	mecC_for: GAAAAAAAGGCTTAGAACGCCTC	(Stegger et al., 2012b)
		717 bp	mecC_rev: CCTGAATCWGCTAATAATATTTC	(Cuny et al., 2011)
cat194	Chloramphenicol	55 °C	catpC194-F: CGACTTTTAGTATAACCACAGA	(Schnellmann et al., 2006)
		650 bp	catpC194-R: GCCAGTCATTAGGCCTAT	(Schnellmann et al., 2006)
cat221	Chloramphenicol	55 °C	catpC221-F: ATTTATGCAATTATGGAAGTTG	(Schnellmann et al., 2006)
		450 bp	catpC221-R: TGAAGCATGGTAACCATCAC	(Schnellmann et al., 2006)
cat223	Chloramphenicol	55 °C	catpC223-F1: GAATCAAATGCTAGTTTTAACTC	(Schnellmann et al., 2006)
		300 bp	catpC223-R: ACATGGTAACCATCACATAC	(Schnellmann et al., 2006)
fexA	Florfenicol	60 °C	fexA_for: GTACTTGTAGGTGCAATTACGGCTGA	(Kehrenberg and Schwarz, 2006)
		1272 bp	fexA_rev: CGCATCTGAGTAGGACATAGCGTC	(Kehrenberg and Schwarz, 2006)
fexB	Florfenicol	55 °C	GM117 fexB for: TTCCCACTATTGGTGAAAGGAT	(Li et al., 2013)
		816 bp	GM118 fexB rev: GCAATTCCCTTTTATGGACGTT	(Li et al., 2013)
spc	Spectinomycin	55 °C	AAD9_for: TGGAAGTTCAATAGTTGGAGTATATC	(Murphy, 1985)
		544 bp	AAD9_rev: CATCTTTCGAGGTAATTTCACCAG	(Murphy, 1985)
spd	Spectinomycin	52 °C	spd_for: CATGAAAATGAAAATTGGTCTTATCC	(Jamrozy et al., 2014)
		317 bp	spd_rev: CCTGTTTCATAAGTTACGATC	(Jamrozy et al., 2014)
spw	Spectinomycin	55 °C	spw_for: ACCATATAATTGACCACAAGCAGC	(Wendlandt et al., 2013)
		630 bp	spw_rev: CAGCCACCTCAGATTCCATT	(Wendlandt et al., 2013)
apmA	Apramycin	60 °C	apmA-fw CGTTTGCTTCGTGCATTAAA	(Fessler et al., 2011)
		656 bp	apmA-rev TTGACACGAAGGAGGGTTTC	(Fessler et al., 2011)
fusB	Fusidic Acid	50 °C	GM073 fusB-F: CCGTCAAAGTTATTCAATCG	(Chen et al., 2010)
		490 bp	GM074 fusB-R: ACAATGAATGCTATCTCGACA	(Chen et al., 2010)
fusD	Fusidic Acid	52 °C	fusD_for: ATGGAAAAACAACTTTACCCTTATC	This work
		639 bp	fusD_rev: TTGAATCTTATTCAAAAAATGATGGAAGTGC	This work

Supplemental Table 1. (continue)

Gene	Resistance phenotype	Annealing temperature Amplicon size	Oligonucleotide primer sequence (5'-3')	Reference
fusC	Fusidic Acid	50 °C	fusCF: GGACTTTATTACATCGATTGAC	(Chen et al., 2010)
		410 bp	GM076fusCR: CTGTCATAACAAATGTAATCTCC	(Chen et al., 2010)
aadD	Gentamicin	60 °C	GM135: TTGGTCGTCAGACTGATGGGCCC	(Argudín et al., 2012)
		462 bp	GM126: CAGTTAAGACCGAAGCGCTCGTCG	(Argudín et al., 2012)
aac(6')/aph(2")	Gentamicin	40 °C	aac-aph_for: GTATTAGAATTTTATGGTGG	This work
		1201 bp	aac-aph_rev: CCATACATTCTTAATATATC	This work
ermA	Erythromycin	48 °C	GM079 ermA for: GCGGTAAACCCCTCTGAG	(Amezaga and McKenzie, 2006)
		433 bp	GM080 ermA rev: GCCTGTCGGAATTGG	(Amezaga and McKenzie, 2006)
ermB	Erythromycin	60 °C	GM081 ermB for: CATTTAACGACGAAACTGGC	(Jensen et al., 1999)
		425 bp	GM082 ermB rev: GGAACATCTGTGGTATGGCG	(Jensen et al., 1999)
ermC	Erythromycin	56 °C	GM083 ermC for: ATCTTTGAAATCGGCTCAGG	This work
		259 bp	GM084 ermC rev: CAAACCCGTATTCCACGATT	This work
tetK/L	Tetracycline	45 °C	GM099 TetKL for: CCTGTTCCCTCTGATAAA	(Pang et al., 1994)
		1030 bp	GM100 TetK rev: CAAACTGGGTAAACACTG	(Pang et al., 1994)
tetM	Tetracycline	45 °C	GM101 tetM for: TATTGGAGTTTTAGCTCATG	This work
		803 bp	GM102 tetM rev: CACTATAAAGGCGTATATATGC	This work

REFERENCES

Amezaga, M. R., and McKenzie, H. (2006). Molecular epidemiology of macrolide resistance in beta-haemolytic streptococci of Lancefield groups A, B, C and G and evidence for a new *mef* element in group G streptococci that carries allelic variants of *mef* and *msr(D)*. *J. Antimicrob. Chemother.* 57, 443–9. doi:10.1093/jac/dki490.

Argudín, M. A., Mendoza, M. C., González-Hevia, M. A., Bances, M., Guerra, B., and Rodicio, M. R. (2012). Genotypes, exotoxin gene content, and antimicrobial resistance of *Staphylococcus aureus* strains recovered from foods and food handlers. *Appl. Environ. Microbiol.* 78, 2930–2935. doi:10.1128/AEM.07487-11.

Becker, K., van Alen, S., Idelevich, E. A., Schleimer, N., Seggewiß, J., Mellmann, A., et al. (2018). Plasmid-Encoded Transferable *mecB* -Mediated Methicillin Resistance in *Staphylococcus aureus*. *Emerg. Infect. Dis.* 24, 242–248. doi:10.3201/eid2402.171074.

Chen, H.-J., Hung, W.-C., Tseng, S.-P., Tsai, J.-C., Hsueh, P.-R., and Teng, L.-J. (2010). Fusidic Acid Resistance Determinants in *Staphylococcus aureus* Clinical Isolates. *Antimicrob. Agents Chemother.* 54, 4985–4991. doi:10.1128/AAC.00523-10.

Cuny, C., Layer, F., Strommenger, B., and Witte, W. (2011). Rare Occurrence of Methicillin-Resistant S*taphylococcus aureus* CC130 with a Novel *mecA* Homologue in Humans in Germany. *PLoS One* 6, e24360. doi:10.1371/journal.pone.0024360.

Fessler, A. T., Kadlec, K., and Schwarz, S. (2011). Novel apramycin resistance gene *apmA* in bovine and porcine methicillin-resistant *Staphylococcus aureus* ST398 isolates. *Antimicrob. Agents Chemother.* 55, 373–5. doi:10.1128/AAC.01124-10.

Jamrozy, D. M., Coldham, N. G., Butaye, P., and Fielder, M. D. (2014). Identification of a novel plasmid-associated spectinomycin adenyltransferase gene *spd* in methicillin-resistant *Staphylococcus aureus* ST398 isolated from animal and human sources. *J. Antimicrob. Chemother.* 69, 1193–1196. doi:10.1093/jac/dkt510.

Jensen, L. B., Frimodt-Møller, N., and Aarestrup, F. M. (1999). Presence of *erm* gene classes in gram-positive bacteria of animal and human origin in Denmark. *FEMS Microbiol. Lett.* 170, 151–8. doi:10.1111/j.1574-6968.1999.tb13368.x.

Kehrenberg, C., and Schwarz, S. (2006). Distribution of florfenicol resistance genes *fexA* and *cfr* among chloramphenicol-resistant Staphylococcus isolates. *Antimicrob. Agents Chemother.* 50, 1156–63. doi:10.1128/AAC.50.4.1156-1163.2006.

Li, J., Shao, B., Shen, J., Wang, S., and Wu, Y. (2013). Occurrence of chloramphenicol-resistance genes as environmental pollutants from swine feedlots. *Environ. Sci. Technol.* 47, 2892–2897. doi:10.1021/es304616c.

Milheiriço, C., Oliveira, D. C., and Lencastre, H. de (2007). Update to the Multiplex PCR Strategy for Assignment of *mec* Element Types in *Staphylococcus aureus*. *Antimicrob. Agents Chemother.* 51, 3374–3377. doi:10.1128/AAC.00275-07.

Murphy, E. (1985). Nucleotide sequence of a spectinomycin adenyltransferase *AAD(9)* determinant from St*aphylococcus aureus* and its relationship to AAD(3";) (9). *Mol. Gen. Genet.* 200, 33–9. doi:10.1007/BF00383309.

Pang, Y., Bosch, T., and Roberts, M. C. (1994). Single polymerase chain reaction for the detection of tetracycline-resistant determinants Tet K and Tet L. *Mol. Cell. Probes* 8, 417–422. doi:10.1006/mcpr.1994.1059.

Schnellmann, C., Gerber, V., Rossano, A., Jaquier, V., Panchaud, Y., Doherr, M. G., et al. (2006). Presence of New *mecA* and *mph(C)* Variants Conferring Antibiotic Resistance in *Staphylococcus spp*. Isolated from the Skin of Horses before and after Clinic Admission. *J. Clin. Microbiol.* 44, 4444–4454. doi:10.1128/JCM.00868-06.

Stegger, M., Andersen, P. S., Kearns, A., Pichon, B., Holmes, M. A., Edwards, G., et al. (2012a). Rapid detection, differentiation and typing of methicillin-resistant *Staphylococcus aureus* harbouring either *mecA* or the new *mecA* homologue *mecALGA251*. *Clin. Microbiol. Infect.* 18, 395–400. doi:10.1111/j.1469-0691.2011.03715.x.

Wendlandt, S., Li, B., Lozano, C., Ma, Z., Torres, C., and Schwarz, S. (2013). Identification of the novel spectinomycin resistance gene *spw* in methicillin-resistant and methicillinsusceptible *Staphylococcus aureus* of human and animal origin. *J. Antimicrob. Chemother.* 68, 1679–1690. doi:10.1093/jac/dkt081.
